# openEHR Archetype Use and Reuse Within Multilingual Clinical Data Sets: Case Study

**DOI:** 10.2196/23361

**Published:** 2020-11-02

**Authors:** Heather Leslie

**Affiliations:** 1 Atomica Informatics Fitzroy Australia; 2 openEHR International Clinical Models Program London United Kingdom

**Keywords:** openEHR, archetype, template, reuse, clinical informatics, COVID-19, standard, crowd sourced, data set, data quality, multilingual, EHR, electronic health record, SARS-CoV-2

## Abstract

**Background:**

Despite electronic health records being in existence for over 50 years, our ability to exchange health data remains frustratingly limited. Commonly used clinical content standards, and the information models that underpin them, are primarily related to health data exchange, and so are usually document- or message-focused. In contrast, over the past 12 years, the Clinical Models program at openEHR International has gradually established a governed, coordinated, and coherent ecosystem of clinical information models, known as openEHR archetypes. Each archetype is designed as a maximal data set for a universal use-case, intended for reuse across various health data sets, known as openEHR templates. To date, only anecdotal evidence has been available to indicate if the hypothesis of archetype reuse across templates is feasible and scalable. As a response to the COVID-19 pandemic, between February and July 2020, 7 openEHR templates were independently created to represent COVID-19–related data sets for symptom screening, confirmed infection reporting, clinical decision support, and research. Each of the templates prioritized reuse of existing use-case agnostic archetypes found in openEHR International's online Clinical Knowledge Manager tool as much as possible. This study is the first opportunity to investigate archetype reuse within a range of diverse, multilingual openEHR templates.

**Objective:**

This study aims to investigate the use and reuse of openEHR archetypes across the 7 openEHR templates as an initial investigation about the reuse of information models across data sets used for a variety of clinical purposes.

**Methods:**

Analysis of both the number of occurrences of archetypes and patterns of occurrence within 7 discrete templates was carried out at the archetype or clinical concept level.

**Results:**

Across all 7 templates collectively, 203 instances of 58 unique archetypes were used. The most frequently used archetype occurred 24 times across 4 of the 7 templates. Total data points per template ranged from 40 to 179. Archetype instances per template ranged from 10 to 62. Unique archetype occurrences ranged from 10 to 28. Existing archetype reuse of use-case agnostic archetypes ranged from 40% to 90%. Total reuse of use-case agnostic archetypes ranged from 40% to 100%.

**Conclusions:**

Investigation of the amount of archetype reuse across the 7 openEHR templates in this initial study has demonstrated significant reuse of archetypes, even across unanticipated, novel modeling challenges and multilingual deployments. While the trigger for the development of each of these templates was the COVID-19 pandemic, the templates represented a variety of types of data sets: symptom screening, infection report, clinical decision support for diagnosis and treatment, and secondary use or research. The findings support the openEHR hypothesis that it is possible to create a shared, public library of standards-based, vendor-neutral clinical information models that can be reused across a diverse range of health data sets.

## Introduction

### Background

Despite electronic health records being in existence for over 50 years, our ability to exchange health data remains frustratingly limited. Semantic interoperability, as defined by the Healthcare Information and Management Systems Society [1], “provides for common underlying models and codification of the data including the use of data elements with standardised definitions from publicly available value sets and coding vocabularies, providing shared understanding and meaning to the user.”

We have many long-established terminologies from which we can draw coded value sets, such as SNOMED-CT (SNOMED Clinical Terms) [2], LOINC (Logical Observation Identifiers Names and Codes) [3], or ICNP (International Classification for Nursing Practice) [4]. Commonly used clinical content standards, and the information models that underpin them, are primarily related to health data exchange, and so are usually document- or message-focused [5]. In contrast to this, there have been two primary efforts to develop standards-based atomic clinical information models—the HL7 [6] Clinical Information Modelling Initiative (CIMI) [7] and the openEHR International [8] Clinical Models program [9]. Each of these groups aims to establish an open and shared library of standards-based, vendor-neutral, and use-case agnostic information models representing clinical concepts.

The vision of creating a public library of information models that potentially hold the whole scope, breadth, depth, and range of the health care domain is, at the very least, rather daunting. It is effectively seeking to establish a governed, coordinated, and coherent ecosystem of health data definitions. The goal is to develop each information model once and reuse them across various health data sets, potentially including those for data exchange, health record persistence, data registries, population health, and research. Due to the novelty of this approach, there is only anecdotal evidence so far on its feasibility.

In response to the COVID-19 pandemic, between February and July 2020, 7 openEHR templates were independently created to represent COVID-19–related data sets for symptom screening, confirmed infection reporting, clinical decision support, and research. Each of the templates prioritized reuse of existing use-case agnostic archetypes found in openEHR International's online Clinical Knowledge Manager [10] (CKM) tool as much as possible.

This case study aims to investigate the use and reuse of openEHR archetypes across the 7 openEHR templates as an initial investigation on the reuse of information models across data sets used for a variety of clinical purposes.

### The openEHR Approach: Archetypes and Templates

Since 2008, the openEHR Clinical Models program has developed a comprehensive and collaborative methodology to develop clinical information models known as openEHR archetypes [11]. It has gradually developed an extensive library of high-quality, multilingual, and use-case agnostic archetypes that can then be aggregated, constrained, and reused in implementable data sets known as openEHR templates.

An openEHR archetype is a computable specification for a single clinical concept, based on the ISO 13606-2 Archetype interchange specification [12]. The archetypes represent clinical knowledge in a consistent, formal, computable format, independent of any software application or technical implementation. Combined with terminology, they provide a standardized and consistent way to capture, store, display, exchange, aggregate, and analyze health data.

The openEHR approach is unique in that an archetype design strategically aims for a notional maximal data set of relevant data elements with a use-case agnostic mindset to support all possible use—the universal use-case. Achieving a complete maximal data set or inclusion of all use cases is impossible to determine, except perhaps with the benefit of hindsight; however, it is the philosophical avoidance of a minimum data set approach that is critical. Best practice in archetype design aims for each archetype to include all data elements useful to express all attributes about the clinical concept, associated metadata describing the concept, use and misuse, and translations from the original authoring language.

Templates represent a specific data set, comprising one or more archetypes that are constrained to accurately match the data set requirements for a particular clinical use case, health domain, profession, or geographical location. The number of archetypes used in a template reflects the required scope of content and level of detail. Some simple templates representing a laboratory test report may comprise only a single archetype. Theoretically, there is no upper limit to the number of archetypes included in a single template. In practice, a consultation note for a first antenatal visit could comprise data elements from 50 or more archetypes to embrace the diversity and detail of clinical information required for an initial pregnancy assessment.

Principles of template development methodology strongly encourage reuse of existing published openEHR archetypes where available, customize existing archetypes to fit the clinical use case, and develop new archetypes only in situations where no previous archetype exists.

The two-level modeling approach described in the openEHR Archetype formalism [13]—defining and standardizing archetypes first, followed by combining and constraining them to create clinical templates—is unique to the openEHR approach. The rigorously governed, published archetypes held in the CKM provide a robust clinical knowledge foundation. Simultaneously, the templates enable modelers to represent diverse and complex real-world clinical information in standardized data sets.

The underlying crowdsourcing approach highlights openness, transparency, and accountability to the openEHR community. The CKM is a critical enabler: an online hub providing a shared library of archetypes and templates; a collaboration portal receiving contributions of models and expertise from the international member community; and a governance tool to manage clinical content publication, language translation, and artifact versioning.

The CKM volunteer community has over 2500 registered users from 103 countries—comprising clinicians, informaticians, software engineers, terminologists, academics, students, and consumers. There are 535 governed archetypes in CKM: 115 of these archetypes have completed peer review and have been published; 26 are currently undergoing a peer-review process of the content; the remainder are candidates for future publication. With an average of 15 data points per archetype, the CKM library equates to more than 8000 data points.

English is the original language of the international CKM, but each archetype can be multilingual. Translation of archetypes is a significant activity by community volunteers. Currently, CKM contains archetypes in 29 languages, with the most common translations into Norwegian Bokmål, German, and Portuguese (Brazil).

### Case Study

In response to the COVID-19 pandemic, several implementers within the openEHR community openly shared their COVID-19–related templates in CKM. It started with one vendor and grew organically into a grassroots, community-driven collaboration. Three phases of template development were identified.

#### Phase 1

In late February, a major Norwegian hospital vendor recognized the need to develop and deploy new software tools in their clinical system to equip clinicians to monitor and report on COVID-19 cases within their hospitals. Within 10 days, two openEHR templates were created and deployed in English and Norwegian Bokmål for use in clinical systems [14,15] in Norway, Slovenia, and the United Kingdom. The templates were uploaded to a CKM COVID-19 public incubator [16] in early March 2020 under a CC-BY license:

- Template 1: Suspected COVID-19 Risk Assessment [17] – based on guidance from advice given by the World Health Organization (WHO) and public health authorities in the United Kingdom, Slovenia, New Zealand, and Norway.
- Template 2: Confirmed COVID-19 Infection Report [18] – replicates a WHO case report form [19].

Due to the rapid deployment priorities imposed by the pandemic, the primary author of templates 1 and 2 developed the templates by reuse of as many existing archetypes as possible and opted to create new COVID-19–specific archetypes to represent the remaining data points (Ian McNicoll, McChB, personal communication). It was a reasonable and pragmatic design decision, with well-understood consequences—effectively a compromise between strategic reuse design principles and speed of modeling.

#### Phase 2

Soon after templates 1 and 2 were uploaded, CKM editors reviewed the COVID-19–specific archetypes. The editors analyzed the requirements from phase 1 and identified the missing archetype concepts in the CKM library. In direct response to the gap analysis, 11 new content-equivalent, use-case agnostic archetypes were created, covering screening questionnaires, travel history, and infectious disease exposure.

Both phase 1 templates were revised and updated using only use-case agnostic archetypes and uploaded to the CKM COVID-19 project [20]:

- Template 3: Suspected COVID-19 Risk Assessment2 [21] – a revised version of template 1
- Template 4: Confirmed COVID-19 Infection Report2 [22] – a revised version of template 2

Also, the modelers created 1:1 mappings [23,24] of all data points from templates 1 to 3 and templates 2 to 4 with a 98% success rate, providing a future migration path should the clinical systems using templates 1 and 2 choose to upgrade to the revised templates and use-case agnostic archetypes.

#### Phase 3

Three more templates were developed in the weeks and months that followed, and these provided an opportunity to test if the new phase 2 archetypes were fit for purpose and able to represent the requirements for other COVID-19–related data sets.

A Chinese university developed an entirely new template, representing the official Chinese Guidelines for Diagnosis and Treatment Guideline of COVID-19 (7th Edition) [25]. This template was used as the basis for a decision support system implemented within a Chinese hospital system in Wuhan, China, deployed in Chinese [26]. It was uploaded to CKM in early April 2020:

- Template 5: COVID-19 Pneumonia Diagnosis and Treatment (7th Edition) [27]

An Italian health software vendor adapted template 3 for a COVID-19 risk screening application with a nephrology focus. First-time modelers developed it for deployment in Italian within Brotzu Hospital, Cagliari, Sardinia [28], and uploaded it to CKM in late April 2020:

- Template 6: Suspected COVID-19 Risk Assessment Nephrology [29]

The Fast Healthcare Interoperability Resources Implementation Guide (FHIR IG) for the German Corona Consensus Data Set (GECCO) [30] supporting COVID-19 research was released in July 2020. In parallel, an openEHR template was developed to replicate the GECCO data set. It was uploaded to CKM in late July 2020:

- Template 7: German Corona Consensus Data Set (GECCO) [31]

## Methods

Analysis of both the number of occurrences of archetypes and patterns of occurrence within 7 discrete openEHR templates was carried out at the archetype or clinical concept level.

### Reuse per Template

The 7 templates were analyzed in terms of:

- Total data points: the total number of data elements or fields represented within a template, which gives an impression of the level of complexity or level of detail within the template;
- Archetype instances: the total number of archetypes used within a template, including reuse;
- Unique archetype occurrences: the total number of archetypes used within a template, excluding any reuse or repetition of archetypes;
- Existing archetype reuse %: the number of use-case agnostic archetypes that existed in CKM before phase 1 that were used in the template, expressed as a percentage of the total number of archetype instances in the template;
- New archetype reuse %: the number of use-case agnostic archetypes that were created during phase 2, used in the template and uploaded to the CKM pool, expressed as a percentage of the total number of archetype instances in the template;
- COVID-specific archetype use %: the number of COVID-19–specific archetypes created during phase 1 that were used in the template, expressed as a percentage of the total number of archetype instances in the template.

### Reuse per Archetype

The 7 templates were analyzed in terms of:

- Archetype reuse: the number of times an archetype was used across all templates;
- Template count: the number of templates that contained at least one occurrence of an archetype.

## Results

### Reuse per Template

The results in Table 1 focus on the overall archetype composition of each template. The three archetype categories in Table 1 are defined as:

- Existing archetypes: archetypes that had been authored before the COVID-19 template was developed and available in CKM;
- New archetypes: archetypes authored as use-case agnostic archetypes during phase 2;
- Phase 1 archetypes: COVID-19–specific archetypes authored during phase 1.

Total data points per template ranged from 40 to 179. The template with the largest number of data points was template 5, the Chinese COVID-19 guideline data set. Archetype instances per template ranged from 10 to 62. The template with the largest number of archetype instances was template 5. Unique archetype occurrences ranged from 10 to 28. The template with the largest number of unique archetypes was template 7, the German GECCO data set. Total reuse of use-case agnostic archetypes ranged from 40% to 100%. Existing archetype reuse of use-case agnostic archetypes ranged from 40% to 90%, and new archetype reuse of use-case agnostic archetypes ranged from 0% to 43%.

COVID-19–specific archetypes created for novel clinical concepts were used in templates 1 and 2. New use-case agnostic archetypes replacing the COVID-19–specific archetypes were used within templates 3, 4, 5, 6, and 7. Template 6 used a combination of ungoverned and new archetypes.

The number of data points per template can be considered a proxy for the level of detail in the template. The number of unique archetypes per template reflects the diversity of clinical content in the clinical requirements and may be considered a proxy for the level of complexity in the template.

**Table 1:** Reuse per template.

Variable		Template						
		1	2	3	4	5	6	7
Total data points, n		60	88	40	102	179	129	124
Archetype instances, n		15	21	16	26	62	10	53
Unique archetype occurrences, n		10	14	16	16	17	10	28
**Total reuse (%)**		40	52	100	100	100	80	100
	Existing archetype reuse, n/N (%)	6/15 (40)	11/21 (52)	13/16 (81)	16/26 (62)	56/62 (90)	5/10 (50)	30/53 (57)
	New archetype reuse, n/N (%)	0/15 (0)	0/21 (0)	3/16 (19)	10/26 (38)	6/62 (10)	3/10 (30)	23/53 (43)
	COVID-19–specific archetype use, n/N (%)	9/15 (60)	10/21 (48)	0/16 (0)	0/26 (0)	0/62 (0)	2/10 (20)	0/53 (0)

### Reuse per Archetype

The results in Table 2 focus on archetype concept reuse within templates by examining how many times each archetype occurs in each template. For example, the first archetype “Laboratory test result” is a published archetype and was used twice in template 2, twice in template 4, 15 times in template 5, and 5 times in template 7, for a total of 24 instances of reuse across 4 templates. Only clinical archetype use within the templates was analyzed.

**Table 2:** Reuse per archetype (total number of archetype instances=203).

Archetype concept name		Publication status^a^	Template							Archetype reuse	Template count
			1	2	3	4	5	6	7		
**Existing archetypes**											
	Laboratory test result	P		2		2	15		5	*24* ^b^	*4*
	Laboratory analyte result	P					15		4	*19*	*2*
	Specimen	P					15			*15*	*1*
	Address	D		3	1	3				*7*	*3*
	Problem/diagnosis	P	1		1	2			3	*7*	*4*
	Health risk assessment	P			1	4		1		*6*	*3*
	Story/History	P	1	2	1	1		1		*6*	*5*
	Body temperature	P	1		1		1		1	*4*	*4*
	Encounter	P	1		1			1		*3*	*3*
	Facility	D		1	1	1				*3*	*3*
	Occupation record	P		1	1	1				*3*	*3*
	Occupation summary	P		1	1	1				*3*	*3*
	Imaging test result	D					2		1	*3*	*2*
	Problem/diagnosis qualifier	P	1		1				1	*3*	*3*
	Dwelling	D			1			1		*2*	*2*
	Living arrangement	D			1			1		*2*	*2*
	Imaging finding	D					2			*2*	*1*
	Report	P		1		1				*2*	*2*
	Service request	P	1		1					*2*	*2*
	Inspired oxygen	P					1		1	*2*	*2*
	Pulse oximetry	P					1		1	*2*	*2*
	Respiration	P					1		1	*2*	*2*
	Symptom/sign	P							1	*1*	*1*
	Age	D					1			*1*	*1*
	Pulse	P							1	*1*	*1*
	Blood pressure	P							1	*1*	*1*
	Differential diagnoses	R					1			*1*	*1*
	SOFA^c^ score	D							1	*1*	*1*
	PaO2/FiO2 ratio	P					1			*1*	*1*
	Tobacco smoking summary	P							1	*1*	*1*
	Exclusion - specific	P							1	*1*	*1*
	Gender	P							1	*1*	*1*
	Clinical frailty score	P							1	*1*	*1*
	Body weight	P							1	*1*	*1*
	Body height	P							1	*1*	*1*
	Resuscitation status –United Kingdom	D							1	*1*	*1*
	Medication summary	D							1	*1*	*1*
**New archetypes**											
	Condition screening questionnaire	D				1	1		12	*14*	*3*
	Symptom/sign screening questionnaire	D			1	2	1	1	1	*6*	*5*
	Management screening questionnaire	D				2	1		3	*6*	*3*
	Travel event	D			1	1		1	1	*4*	*4*
	Medication screening questionnaire	D							4	*4*	*1*
	Overcrowding screening	D			1			1		*2*	*2*
	Exposure assessment questionnaire	D					1		1	*2*	*2*
	Procedure screening questionnaire	D				2				*2*	*1*
	Therapeutic order	D					2			*2*	*1*
	Episode of care	D				1			1	*2*	*2*
	Infectious exposure investigation	D				1				*1*	*1*
**Phase 1 COVID-19–specific archetypes**											
	Symptom/sign – COVID-19	X	6	2						*8*	*2*
	Admission	X		2						*2*	*1*
	Outbreak exposure	X	1	1						*2*	*2*
	Procedure summary	X		2						*2*	*1*
	Travel trip history	X	1	1						*2*	*2*
	Comorbidity summary	X		1						*1*	*1*
	COVID outcomes	X		1						*1*	*1*
	Fever (*Febbre* in Italian)	X						1		*1*	*1*
	Health risk assessment –COVID-19	X	1							*1*	*1*
	Social summary COVID-19	X						1		*1*	*1*

^a^The publication status key is: P=content is published; R=content is undergoing peer review; D=draft candidate; X=ungoverned, specific use-case only.

^b^Important values are italicized.

^c^SOFA: sequential organ failure assessment score.

Across all 7 templates collectively, 203 instances of 58 unique archetypes were used.

There were 48 existing and new use-case agnostic archetypes, of which 26 had completed the peer-review process, and the content published; 1 is currently undergoing the peer-review process; the remaining 21 are draft candidates.

The “Laboratory test result” archetype was the most reused archetype; it occurred 24 times across 4 templates. Reuse across templates reflects the commonality of content, despite different design intents for the templates. The existing “Story/History” and the new “Symptom/sign screening questionnaire” archetypes were reused across the largest number of templates—within 5 out of the 7 templates each.

Many of the existing archetypes were only used once within the context of these 7 templates. These archetypes had been authored for use-case agnostic use before the development of the COVID-19 data sets, so any reuse within these COVID-19 templates demonstrates reusability across both COVID-19 and non–COVID-19 use cases.

Not all archetypes were used in each template, reflecting the diversity of content requirements across the 7 templates.

## Discussion

### Principal Findings

Before February 2020, the focus of openEHR International's CKM was on creating a library of shared archetypes. Templates had been uploaded to CKM, most commonly to demonstrate modeling patterns or to provide exemplars for common types of data sets. Any estimates of reuse of archetypes across templates had been wholly anecdotal, communicated directly by experienced modelers, and ranged from 60% to 90% reuse.

The onset of the COVID-19 pandemic triggered a collaborative openEHR community effort to fast track both archetype and template development, with CKM used as a coordinating hub. The 7 templates uploaded during this time to CKM have provided the first opportunity for a formal analysis of reuse.

Public sharing of the initial templates, templates 1 and 2, included 8 COVID-19–specific archetypes that were necessitated by the novel content combined with rapid implementation deadlines, resulting in relatively low reuse (ie, 40% and 52%, respectively). This was a reasonable and pragmatic modeling decision in the circumstances but diverged from the recommended design philosophy aiming for use-case agnostic archetypes that usually take more time to develop.

Soon after, the CKM editors redesigned the 8 COVID-19–specific archetypes as 11 new use-case agnostic revisions—conceptually equivalent but intentionally designed to allow for broader reuse—and uploaded them as additions to the CKM library. The clinical concepts modeled in the new archetypes included a range of clinical screening question/answer pairs, as well as models for travel history and a risk assessment about exposure to infectious agents. Revised versions of the initial templates, with 100% reuse, were uploaded as templates 3 and 4, along with associated data mappings.

Modeling of questionnaire archetypes had been attempted unsuccessfully in the past, but without success [32]. Driven by the new COVID-19 screening requirements, modelers revisited the challenge of questionnaire modeling. Subsequently, they developed a family of screening questionnaire archetypes that were use-case agnostic and based on an underlying shared pattern, covering the screening for symptoms and signs, conditions, procedures, management and treatment, medication use, and exposure to agents. They were uploaded to a dedicated project in CKM [33] and made available for broader community reuse.

Phase 3 template development provided an opportunity to test and confirm the reuse potential for the new archetypes in additional clinical data sets.

Template 5 represented the official Chinese Clinical Guidance for COVID-19 Pneumonia Diagnosis and Treatment and was implemented as the foundation for a decision support application. This data set was the most extensive and most detailed in terms of both the number of data points and the number of archetype instances. Laboratory and imaging test results triggered system-generated advice about diagnosis and treatment, resulting in high reuse of the “Laboratory test result,” “Laboratory analyte result,” and “Specimen” archetypes. This template achieved 100% reuse of 17 unique archetypes drawn from the “existing” and “new” archetype pools. The archetypes were all translated into Chinese and uploaded to a Chinese equivalent of the CKM tool, known as the Healthcare Modelling Collaboration tool [34].

Template 6 represented Suspected COVID-19 Risk Assessment data within a nephrology context. It was based on template 3, including the screening questionnaire archetypes but reuse was reduced to 80% due to the inclusion of the “Fever” and “Social summary” archetypes intended to meet local data requirements.

Template 7 was created after communication with the authors of the FHIR IG for the GECCO. It was developed to investigate if the clinical content of a data set explicitly developed for implementation in FHIR could also be represented using openEHR archetypes. The resulting template contains the largest number of unique archetypes, which strongly suggests that this template was the most complex of the 7 templates. It was developed in 4 hours and resulted in 100% archetype reuse of 28 unique archetypes drawn from the “existing” and “new” archetype pools. Creation of the template first involved investigation and analysis of the FHIR IG to identify the clinical requirements and archetypes required, followed by aggregation and constraint of each archetype to match the precise requirements of the FHIR data set. Terminology value sets were not included in the modeling as it was assumed that the same value sets in the FHIR IG would be applicable in the openEHR template.

While there is considerable diversity in purpose or intent across the 7 templates, the level of archetype reuse is a clear indication of the level of commonality in the clinical concepts that underpin each data set. In addition, even though the focus and level of detail for each template varied, the shared data models underpinning each template ensured consistency of data across all of them.

It is also important to note the maturity of the archetypes used—70% (26/37) of the “existing” archetypes have completed the content peer-review process and have been published, which may be considered a proxy for data quality. Further investigations about the qualitative and quantitative assessment of archetype quality should be undertaken—firstly to assess each archetype, but also as a proxy for broader data set quality.

In building a template for each new data set, the amount of reuse depends on the similarity of its clinical concepts with archetypes created for inclusion in prior data sets. It is not so much the purpose, level of detail, or complexity of the data set that influences reuse, but rather the commonality of the component clinical concepts that determine which archetypes are required. In practice, each new template developed leverages all prior work that has shaped each existing archetype in the CKM library and, as illustrated by the development of new archetypes for templates 3 and 4, often extends the library collection. The design approach of archetypes as maximal data sets and universal use case for each concept supports the representation of a variety of levels of detail required in data sets. New clinical requirements are added by extending existing archetypes or creating new archetypes for novel concepts. Over time we can expect the number of archetypes to continue to grow and archetype quality enhanced with increasing levels of detail and refinements from the peer-review process. In this context, it is not unreasonable to expect future archetype reuse to remain at similar rates to those demonstrated in this set of COVID-19–related templates.

The 11 new archetypes in phase 2 were strategically designed as draft candidates: aiming for an inclusive, maximal data set about a single clinical concept; intended for a universal use case; discrete in scope, without any overlap with other archetypes.

The current CKM archetype library comprises a range of archetypes used in prior work. Each new, use-case agnostic archetype developed as part of the creation of the COVID-19–related templates added to CKM will be available for reuse in future modeling efforts. In this way, the CKM library will continue to grow, underpinned by technical and editorial governance processes to ensure coordination and coherence of the archetype library.

In this study, we have observed how the collection of archetypes listed in Table 2, a subset of the CKM library, has provided a focused ecosystem of coordinated and coherent information models to underpin each of the 7 data sets. With the whole CKM comprising 500+ archetypes and 8000+ data elements, it becomes more plausible to imagine the potential for this more extensive library of standardized, coordinated, and coherent information models to be able to represent a broader and more diverse range of data sets. In addition, as in the case of the development of template 7, if reuse of archetypes enables the creation of a template comprising 124 data points within 4 hours, the potential time efficiencies gained through archetype reuse is also worthy of further investigation to determine if this is more broadly applicable.

### Conclusion

Investigation of the amount of archetype reuse across the 7 openEHR templates in this initial study has demonstrated significant reuse of archetypes, even across unanticipated, novel modeling challenges and multilingual deployments. While the trigger for the development of each of these templates was the COVID-19 pandemic, the templates represented a variety of types of data sets— symptom screening, infection report, clinical decision support for diagnosis and treatment, and secondary use or research.

The findings support the openEHR hypothesis that it is possible to create a shared, public library of standards-based, vendor-neutral clinical information models that can be reused across a diverse range of health data sets.

Further investigation is strongly recommended to evaluate:

- The realistic extent and scope of a shared library of information models, including the limitations and barriers. Is it plausible to create a single universal health language, or would it be more feasible to develop libraries for specific purposes?
- Clinical knowledge governance requirements for a library of shared information models;
- The measurement of the quality of individual information models;
- The impact on data set quality if based on a foundation of high-quality information models;
- Time and cost efficiencies of creating data sets from a shared library of information models;
- The impact on health data interoperability if shared information models are used as the basis of data exchange directly between clinical systems, in different contexts, and for various purposes;
- The impact on clinical safety when information models are shared and the need for data transformation or mapping is reduced or eliminated;
- The impact on secondary use of data and research if shared information models are used, supporting safe and accurate aggregation and analysis of health data.

## Abbreviations

CIMI: Clinical Information Modelling Initiative
CKM: Clinical Knowledge Manager
FHIR: Fast Healthcare Interoperability Resources
GECCO: German Corona Consensus Data Set
ICNP: International Classification for Nursing Practice
IG: Implementation Guide
LOINC: Logical Observation Identifiers Names and Codes
SNOMED-CT: SNOMED Clinical Terms
WHO: World Health Organization

## References

[1] (2020). Interoperability in Healthcare? What is Interoperability?. HIMSS.

[2] (2020). SNOMED International.

[3] (2020). LOINC.

[4] (2020). International Council of Nurses.

[5] (2020). Interoperability in Healthcare? Standards Development. HIMSS.

[6] (2020). Health Level Seven International.

[7] (2020). Clinical Information Modeling Initiative. Health Level Seven International.

[8] (2020). openEHR International.

[9] (2020). Clinical Models program. openEHR International.

[10] (2020). Clinical Knowledge Manager. openEHR International.

[11] Leslie H, Ljosland Bakke Silje (2017). Peer Review of Clinical Information Models: A Web 2.0 Crowdsourced Approach. Stud Health Technol Inform.

[12] (2019). ISO 13606-2:2019(en) Health informatics — Electronic health record communication — Part 2: Archetype interchange specification. International Organisation for Standardisation.

[13] (2020). Archetype Formalism Overview. openEHR International.

[14] (2020). openEHR Community Rises to the Challenge of Coronavirus. Open Health News.

[15] McNicoll I (2020). openEHR Discourse discussion forums. openEHR COVID-19 Project.

[16] (2020). COVID-19 Incubator. openEHR Clinical Knowledge Manager.

[17] (2020). Suspected COVID-19 risk assessment, draft template. openEHR Clinical Knowledge Manager.

[18] (2020). Confirmed COVID-19 infection report, draft template. openEHR Clinical Knowledge Manager.

[19] (2020). Revised Case Report Form For Confirmed Novel Coronavirus COVID-19. World Health Organization.

[20] (2020). COVID-19 Project. openEHR Clinical Knowledge Manager.

[21] (2020). Suspected COVID-19 risk assessment2, draft template. openEHR Clinical Knowledge Manager.

[22] (2020). Confirmed COVID-19 infection report2, draft template. openEHR Clinical Knowledge Manager.

[23] Mapping spreadsheet for openEHR-Confirmed Covid-19 infection report.v0, draft template. openEHR Foundation, openEHR Clinical Knowledge Manager.

[24] Mapping spreadsheet for openEHR-Suspected Covid-19 assessment.v0, draft template. openEHR Foundation, openEHR Clinical Knowledge Manager.

[25] (2020). Chinese Clinical Guidance For COVID-19 Pneumonia Diagnosis And Treatment (7th Edition). China National Health Commission.

[26] Li M, Leslie H, Qi B, Nan S, Feng H, Cai H, Lu X, Duan H (2020). Development of an openEHR Template for COVID-19 Based on Clinical Guidelines. J Med Internet Res.

[27] (2020). COVID-19 pneumonia diagnosis and treatment (7th Edition), draft template. openEHR Clinical Knowledge Manager.

[28] (2020). COVID-19 App Di Monitoraggio - Software Demonstration. INPECO Webapp Portal.

[29] (2020). Suspected COVID-19 Risk assessment nephrology, draft template. openEHR Clinical Knowledge Manager.

[30] (2020). German Corona Consensus Data Set - Implementation Guide. Simplifier.net.

[31] (2020). German Corona Consensus Data Set (GECCO), draft template. openEHR Clinical Knowledge Manager.

[32] Leslie H (2014). Blog - The Questionnaire Challenge. Atomica Informatics.

[33] (2020). Screening Questionnaire Project. openEHR Clinical Knowledge Manager.

[34] Healthcare Modelling Collaboration. Zhejiang University.

